# Ligature of the Common Iliac Artery for Aneurism—Use of Silver Ligature

**Published:** 1859-10

**Authors:** Warren Stone

**Affiliations:** Professor of Surgery in the University of Louisiana


*Ligature of the Common Iliac Artery for Aneurism—Use of Silver Ligature—Death on the 26th day from Exhaustion, by Dysentery.* By Warren Stone, M. D., Professor of Surgery in the University of Louisiana.

Charles O'Donnell, aged 36 years, was admitted into the Charity Hospital, December 18, 1858, laboring under aneurism of the external iliac and upper part of the femoral arteries. It was a plain unmistakeable aneurismal tumor, extending from two inches below Poupart's ligament, to two inches and a half above it. The tumor was rather prominent, and the pulsation strong, but there was no inflammation or discoloration of the skin. He stated that he first perceived it about the middle of April previous, but he took little notice of it until July, when the leg began to swell below, and he suffered shooting pains down the leg, which disturbed his rest at night. His general health was bad; he could take but little food; stomach much deranged; bowels irregular, sometimes constipated, and sometimes loose, and of a dysenteric character.

As the tumor did not seem to be increasing very fast, it was thought best to give the patient rest, and endeavor to improve his health before resorting to any operation. Under rest, and opiates, the tumor remained nearly stationary for some time, and his general health improved; but about the middle of January, 1859, it suddenly enlarged, and some inflammatory action was set up. The operation by ligature was offered to the patient, as the only chance of saving his life; but as I could not offer him very strong encouragement, he declined having it made. But his sufferings increased, and on the 26th he concluded to take the chance of an operation, which I accordingly made. An incision was made from the upper edge of the tumor nearly in the course of the linea semi-lunaris up to the cartilage of the ribs, and the abdominal walls carefully divided down to the internal common fascia, which was peeled up from its loose attachment to the walls, and with the peritoneum, pushed aside until the artery was uncovered. There was some difficulty in detaching these tissues at the lower part where they came in contact with the tumor and became adherent, and a small rent was made into the peritoneal cavity, but no serious injury was sustained by it. A small portion of omentum protruded once, but it was returned, and did not appear again. A common silk ligature was carried under the common iliac, by means of an aneurismal needle, and the silver wire attached to it and drawn through. This was tied like a common silk ligature and the ends cut near the knot and the points bent down so as not to present to the soft parts. The knot was not drawn strongly as is usual; but only sufficiently so to stop the current of blood in the artery. The wound was brought together by the twisted suture, and the patient appeared in a favorable condition.— The extremity became cooler than the other, and there was some pain in it, but by the use of artificial warmth and diffusible stimulants, the circulation was gradually established, and no symptoms of gangrene appeared.

The tumor subsided very favorably, and the patient was almost entirely relieved from pain in it, and did well for several days, when the dysenteric spmptoms returned with increased violence, and continued until his death, which took place on the 20th February, the twenty-sixth day after the operation. The operation was on the left side, and it is probable that the displacement and handling of the descending colon, and the sigmoid flexure increased the diseased action which already existed.

Being unwell at the time of this patient's death, I did not attend the Hospital for a day or two, and the clerk delivered the body to the wife of the deceased for burial; and no post-mortem examination was made. This was an unpardonable blunder, for it would have been of great value to have observed the manner in which the silver ligature was disposed of, and the result of a moderately light ligature of materials that will not excite ulceration of the coats of the vessel, unless the tissues are completely strangulated. I am confident that the silver thread may be applied sufficiently tight to completely interrupt the current of blood through the artery, without strangulating the tissues of the vessel, so as to cause its division by ulceration. If this is correct, the danger of secondary hæmorrhage in tying large arteries, may be entirely avoided. The danger from hæmorrhage after the ligature for aneurism is so great that it has become the settled practice to use the painful, tedious and uncertain method of compression, where it can be made, and the ligature is only resorted to when this method fails. Setting aside the danger of secondary hæmorrhage, the ligature must be allowed to be the best operation. It is soon completed, and the patient receives immediate relief from pressure and pain in the tumor, which often diminishes in size, and leaves a smaller amount of coagulated blood to absorb than when the circulation is arrested by degrees and the blood in the sack is made to coagulate while it is fully distended. A common silk ligature if applied only just tight enough to the artery to arrest the current of blood, is slow in coming away, and obliterates it as certainly as it it was tied so as to cut the inner coat of the vessel, according to the directions of Jones. When I was a student, my preceptor (the late Dr. Amos Twitchell) made experiments to test the truth of the assertion of this surgeon, that it was necessary to draw the ligature so as to cut the inner coat of the artery in order to produce adhesive inflammation, and proved that it was mere theory, and not a fact. The truth is, that adhesive inflammation does not take place in the inner surface of ligated arteries under any circumstances; at least, I have never found it in the cases I have examined. When an artery is tied in its course, so as to arrest the current of blood, a coagulum is formed on the proximal side, but not on the distal side, of the ligature, and hence it is that hæmorrhage often takes place from the distal extremity, when the anastomosis and collateral circulation is free, in cases when the adhesive process has been perfectly good. The coagulum acts as a plug which is held in place or kept from being forced out by the heart's action, by the lymph that is thrown out in and around its coats so as to thicken them and hold them in the puckered condition in which the ligature places it. On the distal side, as no clot is formed, if the vessel is filled with blood, and the patient makes much exertion about the time the ligature comes away, hæmorrhage will often take place, and I believe is frequently mistaken for hæmorrhage from the proximal side. This mistake may cost the patient his life; for, if a ligature is applied higher or nearer the heart, it may not stop the bleeding, but lead to serious consequences: whereas, a very little compression over the sinus left by the ligature. until the granulations fill it up, renders it perfectly safe.

It is plain, however, that, when an artery is tied in its course, it is not necessary to tie it so as to make a division of its coats by ulceration, if the ligature is of silver or any material that will not cause ulceration by its presence. All that is required of the ligature in the case of aneurism is to arrest the current of blood in the artery, and this can be done without strangulating the tissues inclosed in the ligature so as to cause a division. In the case here relatad, as the vessel tied was so large, and the organic functions were so feebly carried on, I feel confident that hæmorrhage would have taken place if the ordinary silk ligature had been used in the usual way. In this connection I will take occasion to express my sense of what is due to Dr. J. M. Sims, for introducing the silver ligature, as well as establishing a successful method of operating for vesico-vaginal fistula and analogous lesions, which is mainly due to the silver suture.

Up to the time Dr. Sims laid before the profession his mode of operating for vesico-vaginal, it must be admitted that no successful mode of treatment had been established, and hundreds of females laboring under this most loathsome and painful affliction were left in hopeless misery. It is true that an occasional cure was effected under favorable circumstances, but these cases were rare exceptions to the general rule. Since the method of Dr. Sims has been adopted, all the bad cases that were formerly pronounced incurable are cured with great certainty. The attention of the profession was called a few years ago to the button suture of Dr. Bozeman, of Montgomery, Alabama, which, by many, is considered an improvement, and it has been quite generally adopted, It is not my object to discuss the merits of this improvement. Dr. Sims uses the plain interrupted suture, and no one operates with more success, I believe, than he does; but if others realize more favorable results by the button suture, than by the simple, it is their duty to adopt it. I see nothing in this improvement that detracts from the merits of Dr. Sims, however much it may redound to the credit and honor of the inventor. It would seem that Dr. Sims thought otherwise, and setting aside all his well founded claims to merit, for his indomitable perseverance, in inventing instruments and simplifying the operation, he bases them entirely upon the introduction of the silver suture. This he set forth in a paper he read before the New York Academy of Medicine, in which he may have indulged in some pardonable egotism, and unfortunately brought forward personal matters that belonged to him and Dr. Bozeman alone. This gave the friends of Dr. Bozeman (who seemed to have been sharp critics) an excuse for a severe criticism, and they not only ridiculed his paper, but his claim to the discovery of the silver suture. Although there was in these criticisms an affectation of rendering some undefined merit to Dr. Sims, the effect with strangers was calculated to do him great injustice. It was urged (and with truth it must be admitted) that the silver and other metalic sutures were known long since. Dr. Levert, of Mobile, made experiments as long ago as 1828, and published the results (which were highly favorable) in the American Journal of Medical Sciences of that year, but no one acted upon the principle thus established.

When a principle is put to practical use, if it is important, many aspirants spring up for the honor of the discovery. Dr. Simpson, of Edinburgh, on reading the discourse of Dr. Sims, was reminded that he had read somewhere the report of a successful case operated upon in which the silver suture was used. He instituted a correspondence on the subject, and finally found that it was reported in a London journal in the year 1835. Dr. Simpson is a very ingenious man, and ready to adopt any improvement that gives promise of value, but this case passed him unnoticed, and would in all probability, never have been thought of again if he had not read the discourse of Dr. Sims. The remarks of Sidney Smith, in the defence of Hamilton, who established a system of education, when the critics denied the originality of the system, are very appropriate· He says, "whether Hamilton is or is not the inventor of the system that bears his name, or what his claims to originality may be, are questions of very second rate importance; but they merit a few observations That man is not the first discoverer of any art, who first says the thing; but he who says it so long, so loud, and so clearly that he compels mankind to hear him. The man who is so deeply impressed with the importance of the discovery that he will take no denial, but, at the risk of fortune or fame, pushes through all opposition, and is determined that what he has discovered shall not perish for the want of a fair trial. Other persons had witnessed the effect of coal gas in producing light; but Windsor worried the town with bad English for three winters before he could attract any serious attention to his views. Many persons broke stone before Macadam, but Macadam felt the importance of the discovery more strongly, stated it more clearly, persevered in it with greater tenacity, wielded his hammer, in short with greater force than other men, and finally succeeded in bringing his plan into general use."

When Civiale brought before the profession of Paris his admirable surgical operation, called lithotrity, the critics attempted to write him down, and denied that the process of breaking down stone in the bladder was a new invention. After having proved that he was not the inventor of his instruments, they attempted to show that the instruments themselves were detestable, and farther, that Civiale did not know how to use them. Civiale, however, was happy in his associations. The eminent Chaussier and Percy concluded their report upon the subject of Lithotrity, to the Academy of Science, in these words: "Lithotrity is glorious for French surgery, honorable to its inventor, and consoling to humanity;" and a writer, in reply to these critics, who would deprive Civiale of the merit of originality, says : " in effect, the only true proprietor of a surgical improvement is he who applies it successfully, theoretical reasonings and the cavilings of chronologists to the contrary notwithstanding."

Whether Dr. Sims bases his claims for distinction upon his indomitable perseverence under repeated failures and disappointments, ill health and other impediments, in endeavoring to establish the curability of vesico-vaginal fistula, or merely upon the introduction of the silver suture, as embracing the whole merit, he is clearly entitled to it, and these just acknowledgments no more detract from the merit of those who have made valuable improvements, than those improvements detract from the merits of the original inventor.

I have made these few remarks in justice to the profession, rather than from any partiality for any individual member. Whatever an individual member may do that redounds to his honor, sheds a proportionate lustre upon the whole profession, and nothing detracts from it so much as our own bickerings and injustice to each other.

[*N. O. Med. & Surg. Journal.*

